# Synchronous Multiple Parathyroid Carcinoma: A Challenging Diagnosis Influencing Optimal Primary Treatment—A Literature Review to Guide Clinical Decision-Making

**DOI:** 10.3390/jcm14155228

**Published:** 2025-07-24

**Authors:** Emanuela Traini, Andrea Lanzafame, Giulia Carnassale, Giuseppe Daloiso, Niccolò Borghesan, Alejandro Martin Sanchez, Amelia Mattia

**Affiliations:** 1 Endocrine Surgery Unit, Ospedale San Carlo di Nancy-GVM Care and Research, 00165 Rome, Italy; gcarnassale@gvmnet.it (G.C.); amattia@gvmnet.it (A.M.); 2 Department of Medical and Surgical Sciences, Fondazione Policlinico Universitario A. Gemelli IRCCS, 00168 Rome, Italy; 3 Multidisciplinary Breast Center, Dipartimento Scienze della Salute della Donna e del Bambino e di Sanità Pubblica, Fondazione Policlinico Universitario A. Gemelli IRCCS, 00168 Rome, Italy

**Keywords:** synchronous multiple parathyroid carcinoma, primary hyperparathyroidism, parathyroid carcinoma, diagnosis, surgical therapy, management, decision-making

## Abstract

Synchronous multiple parathyroid carcinoma is a rare condition within the already uncommon landscape of parathyroid malignancies, which comprise less than 1% of sporadic primary hyperparathyroidism cases. To date, only seven cases of synchronous multiple parathyroid carcinoma in sporadic primary hyperparathyroidism have been documented. This exceptional rarity complicates both the diagnostic process and therapeutic decision-making. Clinically, parathyroid carcinoma typically presents as a single mass determining severe symptoms. However, no single clinical, biochemical, or imaging feature allows for definitive preoperative diagnosis. Imaging modalities such as ultrasound and sestamibi scans exhibit variable sensitivity and may overlook multi-gland involvement. Histopathological examination remains the only reliable diagnostic method. Management strategies are also controversial: while some advocate for conservative surgery, en bloc resection is generally recommended for its association with improved local control and disease-free survival. Given the exceptional occurrence of synchronous multiple parathyroid carcinoma, there is a lack of standardized protocols for managing parathyroid carcinoma in cases of synchronous and multiple gland involvement. Early multidisciplinary evaluation and individualized treatment planning are therefore crucial. This review aims to synthesize the presently available knowledge about synchronous multiple parathyroid carcinoma, assist clinicians with the limited data available, and discuss the main challenges in the management of this rare entity.

## 1. Introduction

Parathyroid carcinoma (PC) is one of the rarest endocrine malignancies. It typically presents as a solitary, palpable neck mass, often accompanied by severe hypercalcemia and end-organ complications [1,2]. Even more exceptional is the occurrence of synchronous multiple parathyroid carcinoma (SMPC), which poses significant challenges in both diagnosis and treatment. Until 2017, there was no official staging system for PC. The 8th edition of the AJCC Cancer Staging Manual introduced the T, N, and M categories for the first time [3]. However, complete clinical stages have yet to be defined due to the limited data available and uncertainty about the prognostic value of local invasion, lymph node involvement, and tumor size. Despite its rarity, timely recognition and appropriate surgical management are significant determinants of long-term prognosis, and surgical expertise plays a crucial role in ensuring optimal outcomes [2,4]. This review aims to discuss the available knowledge about SMPC. Unfortunately, only a minimal number of cases have been described in the literature until now. Drawing on available literature resources we will discuss the current knowledge, diagnostic challenges, and implications of surgical decision-making in this rare and complex clinical scenario.

## 2. Materials and Methods

This comprehensive review aims to provide a concise yet complete overview of SMPC. We employed a systematic and targeted literature search strategy across indexed databases, including PubMed/MEDLINE (National Library of Medicine), Scopus, and EMBASE. Only studies published in English were included in this review, in order to ensure easy access and full understanding of the sources for all readers, including those who may not have access to advanced translation tools. This choice was made to enhance the overall accessibility and usability of the manuscript. Since SMPC is also known as multiple parathyroid carcinoma (MPC) or double parathyroid carcinoma (DPC) in the literature, all searches were conducted using all these terms. This review included all studies published on the subject.

## 3. Results

From the research conducted, seven studies concerning SMPC were found and included in the review. All the selected articles are case reports and cover the period between 2000 and 2017. Each paper describes only one case. All the cases described were treated at university surgical centers. Demographic, clinical, and laboratory findings are described in Table 1. The published series of SMPC included five male (71.43%) and two female patients (28.57%). The median age was 52 ± 8.18 years (38–67). Six out of seven articles describe the clinical presentation of patients, all of whom were symptomatic [5,6,7,8,9,10]. Among reported cases, the most common symptoms were bone pain (66%), abdominal pain (42%), and fatigue (42%). In two out of six cases (33.3%), the overt symptoms prompted patients to visit the emergency department as the first evaluation [5,10]. In one of them, the patient manifested neuropsychiatric symptoms such as confusion and depression [5]. In the other case, the patient presented at the emergency department with nonspecific symptoms such as dyspnea and tachypnea [10]. In six out of seven cases included in this review, laboratory data at the first patient’s evaluation are reported, specifically concerning calcium and parathyroid hormone (PTH) levels [5,6,7,8,9,11]. In only one case, blood tests were not initially performed because the PC was misdiagnosed as a thoracic mass of other origin [10]. In three out of six patients (50%), the calcium level exceeded 14 mg/dL, with a median value of 14.4 ± 1.9 mg/dL (11.6–17.3), and in three out of six patients (50%), PTH was higher than five times the normal range, with a mean value of 611 ± 449 pg/mL (98–1491).

Details concerning imaging are reported in Table 2. In the reviewed series, imaging was performed on all the patients. However, in the case reported by Dickmen, imaging to localize pathological parathyroid tissue was performed only after the first intervention, which unexpectedly revealed a mediastinal ectopic PC [10]. The choice to perform more than one single localization study was not homogeneous. A sestamibi (MIBI) scan was performed in all cases, and in two out of seven (28.6%), it represented the only preoperative investigation [5,6]. In one out of seven cases, it was performed only after the primary surgery [10]. When performed before primary surgery (six/seven cases), it showed bilateral activity in four out of six cases (66.67%) [5,6,9,11], unilateral activity in one case (16.67%) [8], and was completely negative in one case (16.67%) [7]. Ultrasound (US) was used as the first-line imaging method in five out of seven cases (71.4%) [7,8,9,10,11]. When performed, it was able to detect parathyroid enlargement of only the involved glands in one out of five cases (20%) [9]. In the remaining four cases (80%), US was able to detect only one pathologic parathyroid [7,8,10,11]. In one of these cases, US was unable to recognize one of the parathyroid malignant lesions, misdiagnosing it either as a thyroid nodule or an intrathyroidal parathyroid carcinoma [11]. US features suggested PC in two out of five cases (40%) but never in both the involved glands [7,9]. Only one of the seven cases (14.3%) included in the review involved a patient who received medical treatment designed to reduce calcium levels during the diagnostic workup [7]. Sahasranam et al. described that the patient was treated with adequate hydration for one week, which resulted in improvement in his laboratory tests, showing a decrease in serum calcium to 9.3 mg/dL (from 17.3 mg/dL) [7]. All patients underwent surgical treatment.

Surgical approach, histology, and follow-up data are reported in Table 3. A simple parathyroidectomy (PTX) was carried out in two cases (28.57%) as the first or unique surgical approach [5,6]. Brown et al., described a two-stage surgical approach. During the first intervention, the patient underwent bilateral neck exploration (BNE) and bilateral inferior PTX due to the intraoperative finding of two enlarged glands adherent to the surrounding tissue. After obtaining definitive histology showing PC in both the removed glands, the surgeon opted for a secondary completion thyroidectomy with central neck lymph node dissection (CND) [6]. In the other case, the authors described a double PTX, with no additional notes regarding the intraoperative findings [5]. In these two cases, neither a preoperative nor an intraoperative suspicion of PC was referred by the authors. En bloc resection represented the preferred surgical approach in four cases (57%) [8,9,10,11], in three cases as primary surgery [8,9,11], and in one case as reoperation [10]. In three cases (75%), the decision to perform en bloc resection was indeed guided by intraoperative findings [8,10,11]. In the case reported by Yuan et al., preoperative investigations, including neck US and MIBI scan, indicated unilateral glandular hyperactivity [8]. Despite preoperative single-gland localization, the authors planned a BNE. The intraoperative suspicion of single unilateral PC led to an en bloc resection of the involved parathyroid gland along with the ipsilateral thyroid lobe and adjacent soft tissue. Despite the radicality of primary surgery and the bilateral exploration, postoperative PTH levels remained high, necessitating a second intervention consisting of completion thyroidectomy combined with left inferior PTX, performed three months later. Kamejana et al. described the finding of a markedly enlarged left parathyroid gland, which was removed without the surrounding tissue due to the absence of suspected malignancy. A persistent elevation of intraoperative PTH (Io-PTH) levels led to the identification of the right parathyroid gland, whose appearance was initially uncertain, raising suspicion for either thyroid carcinoma or intrathyroidal PC, and it was removed en bloc with the right thyroid lobe and paratracheal lymph nodes [11]. In the case described by Dikmen et al., the initial chest computed tomography (CT) scan revealed a mass in the anterior mediastinum, which was subsequently removed thoracoscopically. Following the histological diagnosis of PC, the patient underwent further diagnostic investigations and was subsequently treated with en bloc resection of the left superior and inferior parathyroid glands, connected by a fibrous band, along with the left thyroid lobe and left central lymph nodes [10]. Haciyanli et al. instead planned and performed a bilateral en bloc resection of two suspicious PCs [9]. Finally, Sahasranam et al. described bilateral PTX and concomitant total thyroidectomy, but no details about a clear suspicion of PC were reported [7]. Therefore, this case was classified neither as a simple PTX nor as an en bloc resection. Io-PTH monitoring was employed in 2 of the 7 cases included in the review (28.57%) [10,11]. In one case, persistently elevated Io-PTH levels led to a BNE, which identified a second diseased parathyroid gland [11]. In the other case, a 70% Io-PTH drop confirmed the correct identification of the affected parathyroid gland [10]. In one case, the authors specified that Io-PTH was not used because it was unavailable at the time of surgery [7]. Histological examination was performed in all the cases included in this review. Detailed reports are available for six of the seven cases [6,7,8,9,10,11]. In all of these cases, at least one diagnostic criterion for PC is present. Specifically, vascular invasion is reported in five (83.3%), capsular and surrounding tissue invasion in five, and thyroid parenchyma invasion in three of the six cases (50%). In two cases (28.57%), follow-up was not described [5,10]. The mean follow-up registered in the other cases was 23.8 months. In all the cases, patients remained disease-free at the end of the follow-up period. In three of the reported cases, the patient was disease-free, with normal postoperative calcium and PTH levels [8,9,11]. In the other two cases, the patient continued calcium supplementation therapy [6,7]. No cases used genetic tests to exclude association with syndromic forms.

## 4. Discussion

Despite the incidence of PC increasing to 10–13 cases per 10 million people per year over the last few decades [12,13], it remains a very rare entity, accounting for only 1% of sporadic primary hyperparathyroidism (sPHP) [14]. SMPC in a setting of sPHP is exceptional. Only seven cases of sporadic SMPC have been previously reported and they were always documented as isolated experiences at different centers. Given the very limited knowledge available in the literature, we will combine data from existing sources to describe and discuss whether, and how, this exceptional entity diverges from the more ordinary form of PC.

### 4.1. Clinical Presentation

PC presents equally in males and females at a median age of 44–65 years [2]. In published series of SMPC, male sex tends to be more involved than the female counterpart, while the median age of presentation remains similar to that of PC. Unlike the more prevalent types of sPHP linked to adenoma or hyperplasia, PC usually manifests with pronounced signs and symptoms. These include bone and muscle pain, neuropsychiatric issues, kidney stones, hypercalcaemic crises, osteopenia or osteoporosis, pathological fractures, and a noticeable neck mass [1,2]. Also, in the case of SMPC, patients usually presented at diagnosis with overt symptoms such as bone pain, abdominal pain, and fatigue (Table 1). Moreover, sometimes, the predominant symptoms are neuropsychiatric, such as confusion and depression [5]. The severity of the clinical picture and symptoms overlapping with other emergent neurological conditions may lead clinicians to perform first-line imaging with low accuracy for PC and SMPC detection. This may influence both the diagnostic pathway and also the therapeutic approach. Notably, regardless of the clinical presentation, overt symptoms caused by severe hypercalcemia can help clinicians suspect malignant parathyroid disease but are ineffective in distinguishing between single PC and SMPC.

### 4.2. Laboratory Findings

There are no strict boundaries in the literature that clearly separate benign parathyroid disorders from malignant ones. However, PC generally tends to have higher laboratory levels. A serum calcium level exceeding 14 mg/dL and PTH values greater than five times the normal level should raise suspicion of malignancy rather than adenoma [15,16]. Unfortunately, no significant differences in blood tests are registered between single and SMPC. Therefore, laboratory findings, though useful in supporting the PC suspicion, cannot help in suggesting a multi-gland involvement.

### 4.3. Imaging

Imaging investigations of parathyroid lesions are used to precisely locate the affected parathyroid gland before surgery and to distinguish between benign and malignant lesions to determine the optimal surgical approach. US demonstrated a sensitivity of 71%, with a specificity of 100%, in localizing the affected parathyroid gland before surgery [17]. According to the malignancy stratification, US is considered the most reliable imaging method. A tumor size of 30 mm at US is considered a predictive value for the diagnosis of PC in patients with sPHP, with a sensitivity of 91% and specificity of 92% [17]. Both infiltration of surrounding tissue and calcification have a 100% positive predictive value for malignant lesions, while the absence of suspicious vascularity, thick capsule and inhomogeneity have high negative predictive values (97.6%; 96.7%, and 100% respectively) [2]. MIBI scan is the most sensitive localization technique (95%) for identification of pathological glands [18]. In many countries, especially in the USA, MIBI scan is considered the most reliable imaging in sPHP and nuclear medicine is largely used, but its capability in evaluating the risk of malignancy is very low [19]. Both CT and magnetic resonance imaging (MRI) provide valuable insights into the extent of lesions and potential invasion of adjacent structures, lymph node involvement, or distant metastases. However, CT generally exhibits low sensitivity for detecting PC, whereas MRI is more suitable for assessing soft tissue involvement [20]. Combined imaging with US, CT, and MIBI scan achieves 100% sensitivity for localization, whereas single-modality imaging has lower sensitivity. Unlike the ordinary form of single-gland PC, in the case of SMPC, imaging plays a determinant role not only in predicting malignancy but also in suspecting multi-gland involvement. For this reason, the decision to perform more than one single localizing study may significantly impact the correct preoperative diagnosis and reduce the risk of underestimating SMPC. In the reviewed SMPC series, imaging was performed in all patients, but the choice to use more than a single imaging modality was variable. US was not performed in two cases [5,6] reducing the capability to recognize malignant lesions. Concerning the capability of the US to detect more than one gland involvement in SMPC, we noticed that, unfortunately, US was capable of detecting parathyroid enlargement of both the involved glands only in one case [9]. Considering the dimensions of the involved parathyroids found at surgery, it seems quite unreliable that US was unable to detect some of them. They were probably interpreted as thyroid nodules. MIBI scan, instead, showed bilateral activity in more than half of the cases [5,6,9,11]. According to the capability of US to predict parathyroid malignancy, in the reviewed SMPC series, we noticed a drop in the overall accuracy of US when compared to single PC series.

### 4.4. Treatment

Severe hypercalcemia can lead to cardiovascular, neurological, renal, and digestive manifestations. In patients who do not require emergency surgical treatment, the goal of initial clinical management is the reduction of serum calcium levels. The initial treatment for hypercalcemia typically involves intravenous fluids, diuretics, and bisphosphonates, which are intended to lower serum calcium. If these primary therapies prove ineffective, calcimimetic agents such as cinacalcet are recommended. Cinacalcet helps to manage hypercalcemia caused by PC by suppressing PTH secretion; however, its effectiveness may be limited in advanced tumors that do not express the calcium-sensing receptor [18]. In only one of the reviewed cases of SMPC was the initial medical treatment described. In order to avoid an incomplete imaging before surgery, the possibility to lower the serum calcium is of utmost importance to improve the possibility to predict multiple PCs. Surgery serves as the primary treatment for PC and is the only curative option for localized cases [18,21,22]. Preoperative findings seldom suggest malignancy. Nonetheless, intraoperatively, it may be suspected based on the tumor appearance or dissection challenges. If there is a strong suspicion of PC, it is recommended to perform an en bloc tumor removal, ensuring capsule integrity to prevent local spread, along with ipsilateral thyroid lobectomy in the presence of adhesions [18,23]. In the reviewed SMPC series, en bloc resection was performed in four cases, but only in one case did surgeons accomplish it for both the involved glands [8,9,10,11]. The cases included in this review illustrate a markedly heterogeneous therapeutic approach tailored to individual clinical scenarios. This variability reflects, on one hand, the ongoing debates about the best management of PC, and on the other hand, the lack of a clear strategy in the extremely rare occurrence of SMPC. According to the guidelines issued by the European Society of Endocrine Surgeons (ESES), en bloc resection of the affected parathyroid gland together with the ipsilateral thyroid lobe and surrounding soft tissue is recommended in patients with T1 PC [18]. However, this recommendation remains subject to debate within the endocrine surgical community [24]. A recent systematic review published by McInerney et al., which included a total of 2.307 patients, concluded that there is no difference in survival between local resection and extensive radical surgery. Across all studies, PTX alone is the most frequent approach [4]. However, according to an earlier review that included 372 patients, en bloc resection was associated with an 8% local recurrence rate and an 89% long-term overall survival rate (mean follow-up of 69 months) [25]. In contrast, simple PTX resulted in a 51% local recurrence rate and a 53% long-term survival rate (mean follow-up of 62 months). It is worth noting that when only local excision was performed, there was no intraoperative suspicion of malignancy, suggesting that these tumors may have presented at a less advanced stage. Probably most of the SMPCs described in our review that were treated by simple PTX belonged to such a category of PC. Notably, in the reviewed series of SMPC, when en bloc resection was the preferred option, it was accomplished generally for only one of the two involved glands. This suggests either a variable macroscopic appearance of synchronous PC in the same patient or, once again, the influencing weight of the extreme rarity of SMPC on the intraoperative judgement of the surgeon. As described above, primary surgery sometimes failed and required reoperative surgery. In order to reduce the possibility of overlooking multi-gland disease, especially in the case of incomplete preoperative imaging, BNE should always be the preferred option. Io-PTH demonstrated the ability to reduce the risk of persistence in patients undergoing targeted PTX [26,27,28]. Io-PTH was used in two of the seven cases included in the review [10,11]. Unfortunately, no studies specifically addressing the usefulness of Io-PTH in cases of SMPC are available. However, Dobrinjia et al. reported that the use of Io-PTH could serve as a reliable indicator of malignancy during PTX, as malignant cases tend to show higher baseline Io-PTH levels and a more significant drop compared to benign conditions [29]. Moreover, recently, Armstrong et al. showed that a decrease in >50% of Io-PTH into the normal reference range may better predict complete excision of malignant tissue in patients with PC compared to a decrease in >50% of Io-PTH alone [30]. The authors concluded that using Io-PTH with this interpretation criterion in addition to a meticulous surgical technique offers the best results in the treatment of PC. Another important topic of discussion is the indication of whether or not to perform CND in conjunction with en bloc resection of PC. Approximately 10% of PCs show initial lymph node involvement [31]. Currently concomitant neck dissection receives general consensus only in cases with clinical suspicion of lymph node metastases. Insufficient non-radical surgery heightens the risk of recurrence and specific mortality. However, there is no evidence that preventive neck dissection increases survival rates [32]. In summary, currently, no specific guidelines for SMPC are available. Multi-gland involvement and the possibility of presenting as bilateral make the optimal strategy of SMPC more complex than in the case of a usual single PC. First of all, in case of SMPC the risk not to adequately treat the patient at first intervention relies not only on t malignant behavior, as in classical single PC, but also on its multi-gland expression. For this reason, the preoperative study should be particularly detailed and complete. Unfortunately, urgent clinical conditions may represent an obstacle to completing all preoperative studies in favor of an immediate surgical approach. In this sense, it is of utmost importance to pursue a multidisciplinary approach ensuring the best medical treatment before surgery. In any case, if the preoperative studies are incomplete, BNE should be always preferred. Also, the surgical decision-making has peculiar implications, in terms of benefits and risks balance, in the case of SMPC, compared to single PC, especially if it is bilateral. As a matter of fact, in case of a radical surgical approach, the bilateral gland involvement might imply not only total thyroidectomy but also the possibility to add CND and, in some cases, also the possibility to achieve more extensive resection of surrounding tissues, with consequent significantly increased intraoperative risks and potential complications. On the other hand, insufficient radicality at primary intervention may result in the recurrence of disease, and the need of subsequent surgical procedures that are linked to elevated rates of complications [4]. In case of SMPC, surgical expertise is even more crucial than in single PC, in order to obtain the best outcome and reduce complications and recurrence. Indeed, an accurate and expert intraoperative evaluation can guide surgical decision-making to achieve the most radical approach only on the side with evident invasion or in cases of evident lymph node involvement, and to reserve a less extensive surgery if the disease expression is limited and not symmetric. Therefore, in case of suspected SMPC, since the expertise and the availability of intraoperative adjuncts plays a decisive role in optimizing outcomes, the patient should be always addressed to specialized centers.

### 4.5. Pathology and Molecular Analysis

The 2022 World Health Organization (WHO) classification divides parathyroid pathology into three categories: parathyroid adenoma, atypical parathyroid tumor (APT), and PC. From a histopathological perspective, APT and PC share overlapping features. The histological diagnosis of PC still requires the presence of at least one of the following criteria: (i) angioinvasion, defined as tumor infiltration through the vessel wall, with associated thrombus, or intravascular tumor cells admixed with thrombus; (ii) lymphatic invasion; (iii) perineural (intraneural) invasion; (iv) direct malignant invasion into adjacent anatomical structures; or (v) histological or cytological evidence of metastatic disease [33]. The classification criteria for PC remained unclear for a long time; therefore, it is possible that some cases reported in the literature as carcinomas were unrecognized APTs [22]. Histology data available from the cases of SMPC object of the present review are scarce but sufficient to exclude cases of APT. All the cases reported met the criteria for PC diagnosis independently from the clinical expression or intraoperative appearance. From a molecular perspective, there are currently no established clinical guidelines for the routine investigation of genetic mutations in PC. Nonetheless, one of the most thoroughly examined genes in this context is CDC73 (formerly HRPT2), which encodes parafibromin, a tumor-suppressor protein that plays a critical role in transcriptional regulation, cell proliferation, and tumor suppression. Inactivating mutations in CDC73 are often found in both sporadic and syndromic PC, especially linked to hyperparathyroidism–jaw tumor (HPT-JT) syndrome. Other genes associated with PC include NF1, PTEN, and PIK3CA [34]. Molecular sequencing may have clinical value in selected cases, as recent studies have identified potentially effective therapies targeting these molecular alterations [34,35,36].

## 5. Conclusions

The extreme rarity of SMPC cases reported in the literature and the heterogeneous approach described do not allow us to draw definitive conclusions for its best management. However, with the information gathered, it is possible to highlight points to consider that will allow a better approach to this entity. Compared to the classical form of PC, the peculiarity of SMPC relies on multi-gland and potential bilateral involvement. No evident difference in clinical presentation and biochemical findings between classical single PC and SMPC exists that may be used to predict SMPC. Prediction of multi-gland involvement could be improved if complete preoperative localization studies are accomplished. The MIBI scan showed the best accuracy in identifying multi-gland involvement in the reviewed cases, but it did not allow for the identification of malignancy. On the contrary, US proved more effective in detecting malignancy but often failed to identify the second abnormal gland as parathyroid and malignant. If a complete preoperative study is not available before surgery, BNE should always be the preferred surgical option. According to available data, surgical expertise appears to play a crucial role in guiding intraoperative decision-making in cases of SMPC. Indeed, in the case of SMPC compared to single PC, the evaluation of the balance between surgical outcome, risk of complications, and risk of recurrence requires higher expertise to perform tailored surgery. Therefore, SMPC cases, especially in case of reoperation, should always be addressed to referral centers. Finally, as in the case of other rare diseases, all SMPC cases should be collected in international registers and undergo accurate genetic and molecular investigations. Only cumulative and detailed information may allow for better prediction of SMPC in the future and the definition of specific diagnostic and therapeutic guidelines.

## Figures and Tables

**Table 1:** Summary of included studies on the clinical presentation and laboratory findings of SMPC.

Reference	Number of Cases	Age	Sex	Symptoms at Presentation	PTH (pg/mL)	Calcium (mg/dL)
Gray JH, Clin Nucl Med 2000 [5]	1	50	M	Confusion, depression	437	13
Brown JJ, ENT 2002 [6]	1	51	M	Gastrointestinal, bone pain, anxiety	98	14
Kamejana K, Endocr J 2003 [11]	1	67	M	Not described	270	11.6
Sahasranam P, South Med J 2007 [7]	1	53	M	Hematuria, flank/abdominal pain, bone pain, fatigue, nausea, weight loss, enlarging new neck mass	1491	17.3
Yuan S-F, EJSO 2010 [8]	1	38	F	Bone pain, femoral fracture, fatigue, nausea, vomiting, enlarging neck mass	782	16.3
Haciyanli M, Endocr Pract 2011 [9]	1	48	F	Fatigue, joint pain, bone pain	589	14.4
Dikmen K, Pan Afr Med J 2017 [10]	1	57	M	Dyspnea, tachypnea	118.7	11.4

M: male; F: female.

**Table 2:** Summary of included studies on the imaging investigations of SMPC.

Reference	Type of Center	Ultrasound Findings	MIBI Scan Findings	Radiological Suspicion of PC	Number of Glands Involved
Gray JH, Clin Nucl Med 2000 [5]	University center	Not reported	Bilateral inferior	Not declared	2
Brown JJ, ENT 2002 [6]	University center	Not reported	Bilateral inferior	Not declared	2
Kamejana K, Endocr J 2003 [11]	University center	Both detected but one suspicious for PTC or PC	Bilateral inferior	Doubtful, only one	2
Sahasranam P, South Med J 2007 [7]	University center	Only one was detected and suspicious for PC	Negative	Yes	2
Yuan S-F, EJSO 2010 [8]	University center	Only one detected	Only right	Not declared	2
Haciyanli M, Endocr Pract 2011 [9]	University center	Both were detected, but only one was suspicious for PC	Bilateral	Yes	2
Dikmen K, Pan Afr Med J 2017 [10]	University center	Only one detected (performed after 1st intervention)	Only right (performed after 1st intervention)	No	2

PC: parathyroid carcinoma; PTC: papillary thyroid carcinoma.

**Table 3:** Summary of included studies on surgical treatment, histology, and follow-up of SMPC.

Reference	Emergency Surgery	Type of First Intervention	Need for Completion	Intraoperative Suspicion of PC	Histology PC Criteria	Genetic Testing	Follow-Up (Months)
Gray JH, Clin Nucl Med 2000 [5]	Not specified	Double PTX	No	Yes	Yes	No	-
Brown JJ, ENT 2002 [6]	No	BNE, double inferior PTX	Yes	No	Yes	No	27
Kamejana K, Endocr J 2003 [11]	No	Left PTX + Io-PTH, en-bloc resection of upper right parathyroid + right thyroid lobe	No	Yes, only on the right side	Yes	No	8
Sahasranam P, South Med J 2007 [7]	No	Total thyroidectomy + PTX (not specified)	No	Not specified	Yes	No	24
Yuan S-F, EJSO 2010 [8]	No	En-bloc resection of right parathyroid and thyroid lobe with surrounding tissue	Yes	Yes, only on the right side	Yes	No	12
Haciyanli M, Endocr Pract 2011 [9]	No	BNE, en-bloc resection of right parathyroid gland + right lobectomy + adherent soft tissue + lower left PTX en-bloc with surrounding tissue	No	Yes, upper right and lower left	Yes	No	48
Dikmen K, Pan Afr Med J 2017 [10]	Yes	en bloc resection of double left parathyroids + left thyroid lobectomy, left CND	Yes	No	Yes	No	-

PC: parathyroid carcinoma; Io-PTH: intraoperative PTH; PTX: parathyroidectomy; BNE: bilateral neck exploration; CND: central neck dissection.

## Abbreviations

The following abbreviations are used in this manuscript:

PC	Parathyroid carcinoma
SMPC	Synchronous multiple parathyroid carcinoma
US	Ultrasound scan
MIBI	Sestamibi
MPC	Multiple parathyroid carcinoma
DPC	Double parathyroid carcinoma
CT	Computed tomography
sPHP	Sporadic primary hyperparathyroidism
PTH	Parathyroid hormone
PTX	Parathyroidectomy
BNE	Bilateral neck exploration
CND	Central neck dissection
Io-PTH	Intraoperative PTH
MRI	Magnetic resonance imaging
ESES	European Society of Endocrine Surgeons
WHO	World Health Organization
APT	Atypical parathyroid tumor
HPT-JT	Hyperparathyroidism–jaw tumor
